# The Impact of Mutant p53 in the Non-Coding RNA World

**DOI:** 10.3390/biom10030472

**Published:** 2020-03-19

**Authors:** Silvia Di Agostino

**Affiliations:** IRCCS Regina Elena National Cancer Institute-IFO, Oncogenomic and Epigenetic Unit; via Elio Chianesi, 53-00144 Rome, Italy

**Keywords:** mutant p53, gain-of-function, long non-coding RNA (lncRNAs), circular RNA (circRNAs), micro RNA (miRNAs), extracellular RNA (exRNAs), cancer

## Abstract

Long non-coding RNAs (lncRNAs), circular RNAs (circRNAs), micro RNAs (miRNAs), and extracellular RNAs (exRNAs) are new groups of RNAs with regulation activities that have low or no protein-coding ability. Emerging evidence suggests that deregulated expression of these non-coding RNAs is associated with the induction and progression of diverse tumors throughout epigenetic, transcriptional, and post-transcriptional modifications. A consistent number of non-coding RNAs (ncRNAs) has been shown to be regulated by p53, the most important tumor suppressor of the cells frequently mutated in human cancer. It has been shown that some mutant p53 proteins are associated with the loss of tumor suppressor activity and the acquisition of new oncogenic functions named gain-of-function activities. In this review, we highlight recent lines of evidence suggesting that mutant p53 is involved in the expression of specific ncRNAs to gain oncogenic functions through the creation of a complex network of pathways that influence each other.

## 1. Introduction

### 1.1. Mutant p53 Gain-of-Function

The p53 protein is a homotetrameric transcription factor whose main function is to act as a tumor suppressor regulating the transcriptional program of downstream target genes [1,2]. The expression of p53 protein is subject to a fine and rapid regulation in the cells. Its levels are low in normal cells and tissues under non-stressed conditions having a very short protein half-life, but its expression is suddenly increased by a diverse range of extra- and intra-cellular stress signals, including DNA damage, oncogene activation, hypoxia, and ROS (reactive oxygen species) [1,2]. The sequencing of human cancer genomes identifies a plethora of genomic and chromosomal mutations in a lot of human cancers. According to the TCGA (The Cancer Genome Atlas) records, more than 50% of all cancers express mutant p53 (mut-p53) proteins reaching levels higher than 90% in small-cell lung cancer and ovarian cancer [3,4]. The relevance of p53 in the suppression of cancer is evident due to the high rate of p53 mutations in all cancers, which is associated with poor prognosis of patients, early onset and frequency of cancers in Li–Fraumeni disease, and great number of mouse models that show cancerogenesis in p53 lost/mutation conditions [5,6,7]. 

Mutant p53 loses tumor suppressive functions and in addition gains tumor-promoting activities that are called gain-of-function (GOF) activities [8,9]. Efficient mut-p53 GOF activity requires high mut-p53 protein expression levels in the cancer cell. In fact unlike wild-type p53 protein (wt-p53), mut-p53 is a rather stable protein due to different mechanisms [5,8,9]. Approximately 70% of *TP53* mutations are missense in one allele with loss of the second allele by loss of heterozygosity (LOH). Many mut-p53 proteins have dominant-negative (DN) effect on the wt-p53 allele. However, the formation of heterotetrameric mut-p53/wt-p53 complex can inhibit the function of the remaining wt-p53 in tumor suppression [8,9]. Most of the missense mutations occur in the p53 DNA-binding region and can be classified as either contact mutations (as p53R248 and p53R273 interfere directly with DNA binding) or conformational mutations (as p53R175 induces local or global conformational distortions) [5,9].

Six hotspot mutations are the most represented in the cancers. These include R175, G245, R248, R249, R273, and R282, which make up about 30% of all mutations in *TP53* covering all human cancer types [8,9,10]. However, due to cancer genome sequencing tools, many other different *TP53* mutations have been discovered. mut-p53 GOF has been demonstrated by numerous cell-based experiments such as by ectopic expression of mut-p53 proteins in p53-null human tumor cells or knockdown of endogenous mut-p53 in cells containing only one allele of mutant p53, as well as in mutant p53 knock-in mouse models [5,8,9,10]. Genome sequencencing has highlighted that more than 91% of *TP53*-mutant cancers exhibit loss of the second allele (LOH) by mutation or DNA deletion [11].

**Figure 1:**
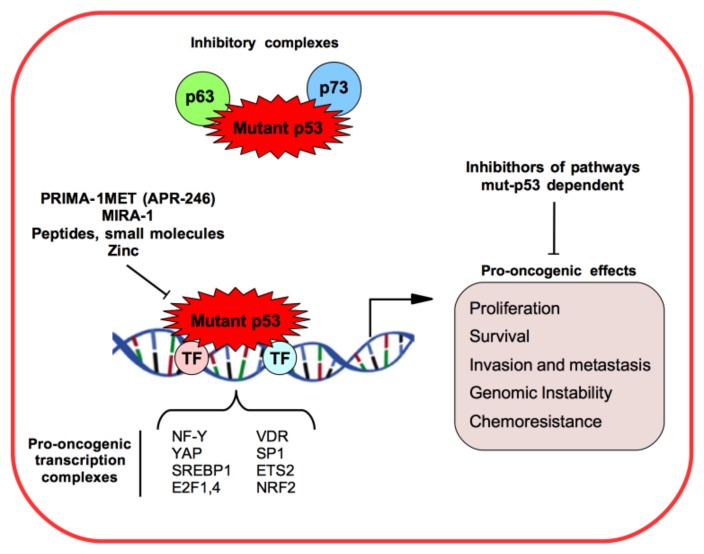
Mutant p53 (mut-53) gain-of-function mechanisms and pro-oncogenic effects in cancer models.

Many mut-p53 GOF activities have been identified as tumor cell proliferation, survival, migration and invasion, enhancing chemoresistance, disrupting proper tissue architecture, inducing cancer metabolism (Warburg effect and lipid metabolism), and increasing genomic instability and mitochondrial dysfunction [5,10,11,12,13,14,15] (Figure 1). 

The mut-p53 protein does not recognize and bind to p53 DNA binding consensus elements and it loses the transcriptional activity towards wt-p53-regulated target genes [8,9,10]. However, mut-p53 proteins are able to aberrantly regulate the transcription of some genes through different mechanisms from wild-type p53, as binding other transcriptional factors and cofactors practically parasitizing their binding sequences onto the target gene promoters [8,9,10] (Figure 1). For example, mut-p53 can interact with NF-Y, SREBP, VDR, Sp1, ETS2, E2F4, YAP, and NRF2 transcription factors, and enhance or decrease their transcription activities to promote tumor progression and spread [8,9,10,11,12,15,16,17,18,19] (Figure 1). 

The other two members of the p53 family, p73 and p63 transcriptional factors, have 22–29% of homology in the transactivation domain (TAD), 63% in the DNA binding domain (DBD), and 42% in the oligomerization domain (OD) of p53 [19]. Both p73 and p63 can promote p53-independent DNA damage response (DDR), growth arrest, and apoptosis [20]. Human tumor-derived p53 mutants are observed to bind diverse p73 and p63 isoforms interfering with their transcriptional activity and inhibiting apoptosis induction and increasing chemoresistance mechanisms (Figure 1) [20].

Among the mut-p53 GOF activities recently discovered, we can include the contribution of mut-p53 to metabolic regulation in different types of cells and tissues, as well as in response to different stress signals, such as glucose starvation, DNA damage, and oncogene activation. [15]. 

Of great interest for several years was the study of peptides and small molecules that aim to stabilize mutant p53 in its physiological conformation, thus restoring its DNA-binding capability and hypothetically rescuing wild-type p53 activity (i.e., DNA damage repair, DNA damage response, cell-cycle checkpoints, apoptosis, and autophagy) [21,22,23]. Currently, the zinc metallochaperones (ZMCs), a new class of mutant p53 reactivators, are being studied [24,25]. The p53 tumor suppressor contains a single zinc ion (Zn^2+^) near its DNA binding domain which contributes to the correct folding of the protein. It was reported that some mutant p53 proteins, as R175H mutant, fail to bind the zinc ion resulting in misfolding of the protein [25]. The zinc metallochaperones could reactivate mutant p53 restoring the wild-type conformation by reestablishing zinc binding and activating p53 through post-translational modifications [24,25]. 

Other lines of research are trying to target downstream oncogenic pathways found to be deregulated and dependent by mutant p53 GOF activities [23,26]. 

The small molecules PRIMA-1 and MIRA-1 have been identified by a cell-based screen of two thousand compounds from the National Cancer Institute (NCI) library as targeting p53 mutant forms of p53 and restoring wt-p53 transcriptional activity and having as readout the cell cycle arrest or apoptosis of tumor cells (Figure 1) [27]. Unfortunately, MIRA-1 was not further addressed due to high toxicity while PRIMA-1MET (APR-246) has shown strong cytotoxic and apoptotic effects in murine cancer models. APR-246 is a molecule that is converted to the active compound methylene quinuclidinone (MQ), a Michael acceptor that binds to Cys124 amino acid in a pocket between loop L1 and sheet S3 of the p53 core domain, restoring its wild-type conformation [28]. The most accredited mechanism of action seems to be that this binding restores a correct folded p53 protein with the consequent rescue of the associated transcriptional activity wild-type p53 [28].

Currently, to test APR-246 efficacy, patients carrying mutations in p53 are enrolled in diverse clinical studies of phases Ib/II as metastatic esophageal or gastro-esophageal junction cancers (NCT02999893), high-grade serous ovarian cancers (NCT02098343 and NCT03268382) or myeloid neoplasms (NCT03072043) [27].

### 1.2. Non-Coding RNAs in Cancer

In the last 30 years, many studies have described different types of RNA including non-coding RNAs (ncRNA) that are not involved in producing proteins, and have proven to play key regulatory roles in shaping cellular activity. Whole genome sequencing revealed that about 90% of the eukaryotic genome is transcribed. Interestingly, only 1–2% of these transcripts encode for proteins, the rest is transcribed as ncRNAs [29,30]. Accordingly, many ncRNAs have shown to be involved in both normal cellular function and disease, including cancer, becoming important targets in the clinical setting. Some ncRNAs are so stable that they survive in the bloodstream so that in cancer patients, a little sample of blood could be used to look for the presence of ncRNAs which is useful in making diagnoses, follow the tumor course, and discovering any recurrence or metastases at early stages [31,32,33].

Importantly, ncRNA can be therapeutical targets as shown by the delivery of oligonucleotides (si-RNAs) targeting protein-coding messenger RNAs (mRNAs) [33,34]. In reference to this topic, the FDA has recently approved the first RNAi drug Onpattro (patisiran), which reduces levels of Transthyretin or TTR for the treatment of the neurodegenerative disease hereditary transthyretin amyloidosis [34,35] and the RNA-targeting oligonucleotide drug Spinraza (nusinersen) which increases levels of full-length Survival of motor neuron 2 or SMN2 for the treatment of the neuromuscular disease spinal muscular atrophy [36]. 

Non-coding RNAs are classified as microRNAs (miRNAs), long non-coding RNAs (lncRNAs), circular RNAs (circRNAs), ribosomal RNAs (rRNAs), transfer RNAs (tRNAs), and PIWI-interacting RNAs (piRNAs). Most of these were discovered by the ENCODE project (Encyclopedia of DNA Elements; https://www.encodeproject.org/) and many of the functions remain unknown, but it is now ascertained that among them are the creation of intricate and complex networks of cellular signaling both in healthy cells and in diseases [29,37,38].

In this review, we aim to discuss the direct and indirect relationships between mutant p53 proteins and ncRNAs that have been highlighted in various types of cancers during important mechanisms of tumor progression and metastasis. Although the importance of piRNAs and tRNAs in cancer has been increasingly recognized, to date any studies explored their eventual crosstalk with mutant p53 expression or activities in cancer development and progression [39]. 

It will be evident that targeting mut-p53/ncRNA signaling may have great potential for impacting cancer patient care.

## 2. Mutant p53 and miRNAs

Mature miRNAs are short, single-stranded ncRNAs of about 21–25 nucleotides in length whose main function is to regulate the levels of other RNAs, most frequently by binding them in a specific complementary sequence (called “seed” sequence) to the 3’ untranslated regions (3’-UTR) in the RNA targets inhibiting their use by either degradation or translational repression. miRNAs can also bind coding regions, 5′-UTR, and open reading frames (ORF) regulating in this way the protein translation. Genes encoding miRNAs are transcribed by RNA polymerase II (Pol II) and processed from the nucleus to the cytoplasm through an evolutionarily-conserved pathway [40,41]. To date, over 2000 miRNAs have been discovered in humans and they collectively may regulate one third of the mRNAs [40,41,42]. MicroRNAs have been linked to many human diseases and are potentially important targets for the diagnostics and therapy. Due to the imperfect complementarity needed for binding target mRNAs, miRNAs have the potential to target hundreds of mRNAs that can fit both tumor suppression and oncogenesis—gene expression, protein regulation, homeostasis, and diseases [40,41,42].

Wild-type p53 (wt-p53), as principal tumor suppressor in the cells, can modulate the expression of many miRNAs [41,43]. Usually, wt-p53 regulates miRNAs to suppress cancerogenesis, on the other hand GOF mut-p53 directly or indirectly modulates microRNAs by conferring oncogenic properties such as chemoresistance and invasion. Therefore, in connecting the miRNA pathway with the mut-p53-regulated pathways it is important to dissect key oncogenic mechanisms that could be druggable. Furthermore, the expression of both wt- and mut-p53 is closely regulated by a fine-tuned machinery including miRNAs. miRNAs directly target *p53* RNA or other factors in the p53 network so that expression and function of either the wild-type or mutant p53 proteins are down-regulated [41,43]. 

Recent studies have explored the correlations between cancers and miRNA signatures as a potential tool for diagnosis and outcome predictions [11,44,45]. The miRNA expression in tumors may be influenced by multiple factors and mutational events, such as gene copy number alteration and transcription process dysregulation. In this scenario also, mut-p53 plays a role in affecting the expression of miRNAs [11]. It is important to evaluate whether the mut-p53-dependent miRNA signature may be prognostic in cancers. A recent study analyzed *TP53* mutations in whole exome sequences from TCGA oncologic patients (10225) across 32 different tumor types. The result was that mutant p53 RNA expression signature was involved in the prognostic predictions in 11 different cancers [11]. Therefore, miRNAs participate in the suppression or induction of tumor development depending on the wt- or mut-p53 cell context [11,46]. 

However one of the most important contributions of the scientific community is studying the mechanisms of transcriptional regulation of miRNA expression by mut-p53 proteins to gain oncogenic functions as well as identifying a gene network regulated by the miRNAs downstream of mut-p53. 

### 2.1. miRNAs Induced by Mutant p53

mut-p53 affects miRNA expression by inhibiting those which play a tumor-suppressing role and inducing those which have oncogenic potential [46]. 

The miRNAs that are up-regulated by mut-p53 include miR-155 and miR-128-2 in breast cancer whose up-regulation results in increased cell proliferation, epithelial-mesenchymal-transition (EMT) and invasion (Table 1) [47,48]. As GOF *TP53* mutations are associated with tumors under high replicative stress, high genomic instability, and reduced patient survival, it was very recently reported that mut-p53, by the induction of miR-205-5p expression, is able to repress the expression of genes involved in DNA repair of DNA double-strand breaks in head and neck squamous cell carcinoma (HNSCC) (BRCA1 and RAD17) [49]. This mechanism leads to inefficient DNA repair and increased chromosomal instability. 

Mutation in the TP53 gene is associated with a low survival in HNSCC patients [50,51]. In HNSCC patients, miRNAs whose expression is associated with diverse GOF TP53 mutations were identified [44]. A miRNA signature composed of 12 miRNAs whose expression correlated with recurrence-free survival and a smaller signature of four miRNAs with cancer-specific survival were selected. Importantly, miRNAs that correlated with survival were independent prognostic factors either when considered individually or as signatures [44]. Among the 12 miRNA signatures, miR-205-5p had the best score in predicting the risk of recurrence in HNSCCs [44,49]. Furthermore high miR-205-5p expression in HNSCC peritumoral tissues was relevant for the early detection of minimal residual disease involved in tumor development [49]. These works are examples where the expression of a miRNA signature was common to different p53 mutations with GOF activities but within only one type of cancer. This strongly suggests that in HNSCC, miRNA signatures could be useful for diagnosis and prognosis in association with the knowledge of *TP53* status [44]. The biological activity of miRNAs depends on the tissue context and the mutational background. In fact, in other tumors miR-205-5p has been shown to act as a tumor suppressor [52].

Other studies have instead correlated miRNA signatures with specific *TP53* mutations and then analyzed these in different types of cancer as tools that may be useful for patient outcome. In an unbiased manner, a signature of miRNAs was found to be associated with mut-p53R282W protein [53]. In breast, liver, and gastric cancer bearing this p53 mutation, this signature has been shown to be significantly associated with disease-free survival and patient prognosis [53].

A novel role of mut-p53 is correlated with the tumor-associated macrophage activity (TAM), a hallmark of solid tumors, which is typically correlated with poor prognosis [54]. Cooks and colleagues described a non-cell-autonomous mechanism, where human mut-p53 cancer cells reprogram macrophages to a tumor-supportive and anti-inflammatory state in colon cancer models [55]. 

Gain of function mut-p53 cells selectively release exosomes enriched with miR-1246. Absorption of these exosomes by neighboring macrophages induces their miR-1246-dependent reprogramming in a cancer-promoting state. The TAMs reprogrammed by mut-p53 promote anti-inflammatory immunosuppression with increased TGF-β activity. These findings support the microenvironmental role of mut-p53 in the active involvement of the immune system to promote cancer progression and metastasis [55].

### 2.2. miRNAs Inhibited by Mutant p53

By inhibitory transcriptional activity, mut-p53 can inhibit tumor-suppressive miRNAs (Table 2). 

miR-34 has been shown to be poorly regulated in diverse cancers, and it also was the first miRNA demonstrated to be directly up-regulated by wild-type p53 in the onset of cancerogenesis [56]. Thus the miR-34 family has been shown to inhibit tumorigenesis. In ovarian and lung cancer, human and mouse models expressing mutant p53, a global down-regulation of miR-34 family expression, also depending on the tumor stage was reported [57,58]. Delivery of ectopic miR-34 in mut-p53 cell context recovered tumor suppressive activities and resulted in cell apoptosis via inhibition of anti-apoptotic genes [57,58]. This evidence points to the great amount of research surrounding ‘miRNA replacement therapy’, that is, the re-introduction of miRNAs suppressed in p53-mutant cancer cells to reactivate cellular pathways initiating a therapeutic response [59]. This provides an introduction into synthetic miR-34 or miR-34 mimetics in pathological tissues in an effort to rescue normal proliferation, apoptosis, and other cellular functions [59,60]. 

To date no clinical trials are underway and some research groups are testing the combination of miRNA-based therapy with other anti-cancer therapies (miRNA-based combinatorial cancer therapy) [61]. This approach is an attractive perspective for intervening in the event that a tumor manifests the acquired therapeutic resistance which leads to cancer relapse. miRNAs are able to target several genes associated with the signaling pathways controlling therapeutic resistance. miRNA-based combinatorial cancer therapy might overcome the therapeutic resistance present in different types of cancer [61].

It has been shown that mut-p53 proteins down-regulate miR-223 expression in breast and colon cancer cell lines [62]. Mutant p53 forms a complex with the transcriptional factor ZEB1, and together they bind the miR-223 promoter reducing its transcriptional activity [62]. The authors have validated stathmin-1 (STMN-1) as a miR-223 target; STMN-1, a protein with oncogenic activity, has been shown to confer resistance to chemotherapeutic agents associated with poor clinical prognosis [62]. This study highlighted that one of the pathways affected by mut-p53 to increase cellular resistance to chemotherapeutic agents involved miR-223 down-regulation and the consequent up-regulation of STMN-1 [62]. Recent studies investigated the role of miR-223-3p in lung squamous cell carcinoma (LSCC) xenografts [63]. Luo and colleagues reported that miR-223-3p was down-regulated in LSCC tissues that formed xenografts (XG) compared to tumor tissues that instead fail. It was also significantly reduced in LSCC tissues compared with the adjacent normal tissues. Importantly, this study showed that mut-p53 bound to the promoter region of miR-223 in vivo, reducing its transcription. Moreover, *p53* RNA was a direct target of miR-223-3p. This feedback loop markedly inhibited cell proliferation and migration [63].

In breast cancer patients, significantly decreased let-7i levels were associated with missense mut-p53 [64]. 

Functionally, the repression of let-7i expression by mut-p53 enhanced migration, invasion and metastasis, and induced a network of oncogenes including E2F5, LIN28B, MYC, and NRAS [64]. Very recently it was reported that reduced miR-30c expression was tightly correlated with human breast cancer and with mutational status of *TP53* gene and associated with low survival [65].

It has been shown that mut-p53 increased intrinsic resistance to chemotherapies down-regulating miR-30c with the consequent up-regulation of DNA repair protein, Fanconi anemia complementation group F protein (FANCF), and the translation synthesis DNA polymerase REV1 protein, two factors that are frequently abundant in the context of mut-p53-breast cancer [65]. 

More recently it was reported that Drosha and Dicer, two key enzymes of miRNA processing, are targets of miR-128-3p which is up-regulated in lung cancer tissues [66]. This leads to a global down-regulation of miRNA expression contributing to the tumorigenic properties [66]. In particular, miR-128-3p-mediated miRNA down-regulation contributed to aberrant control of migratory phenotype of lung cancer cells [66].

Recent evidence highlights the role of mut-p53 also in the post-transcriptional control of miRNA maturation. Mutant p53 has been published to decrease the protein expression level of Dicer [67]. Furthermore, it has been found that diverse GOF mut-p53 proteins sequester the p72/p82 subunits of the Drosha/Microprocessor complex, negatively affecting a group of miRNAs carrying out tumor suppressive activities [68].

## 3. Mutant p53 and Long Non-Coding RNAs

Long non-coding RNAs (lncRNAs) are non-coding transcripts greater than 200 base pairs in length transcribed by RNA Pol II from independent promoters. Similar to protein-coding genes, next generation sequencing by ChIP experiments highlighted that their genomic loci are characterized by enriching H3K4 trimethylation at the transcriptional start site and H3K36 trimethylation throughout the entire gene [69]. The lncRNA transcripts are spliced through conserved mechanisms into a mature transcript, they have fewer exons and are expressed at lower levels overall compared to protein-coding transcripts [69]. On the basis of the ENCODE project, it is estimated that the human genome encodes more than 30,000 distinct lncRNAs, many of which are still being found and are yet to be annotated [70]. Functionally, lncRNAs are classified as signaling, decoyed, guided, and scaffolded lncRNAs [71]. Signaling lncRNAs are associated with specific signaling pathways, decoyed lncRNAs interact with and titrate away proteins or other RNAs, guided lncRNAs bind to the regulatory or enzymatically-active protein complexes and direct them to specific target gene promoters regulating downstream pathways and gene expressions, scaffolded lncRNAs act as a platform to which different kind of protein complexes [71]. The lncRNAs play key roles in regulating chromatin structure, gene expression, cell growth, differentiation, and development and their mutations or dysregulation of their expression are associated with a wide range of diseases, especially tumors and neurodegenerative diseases [72,73].

LincRNA-p21, together with other lncRNAs, was the first lncRNAs identified to be transcriptionally induced by wt-p53 (Table 3) [74]. It served as a transcriptional repressor in the p53 pathway. The authors showed that lincRNA-p21 was able to trigger growth arrest and apoptosis through binding with hnRNP-K [74]. 

After this work, several other lines of research have correlated lncRNAs with the expression of wt-p53 in different human cancers whereas up to today few papers have documented the relationship between mutant p53 and lncRNA expression (Table 3) [75,76]. 

Of considerable interest, MALAT1, also known as nuclear-enriched abundant transcript 2 or alpha, is a broadly expressed lncRNA with a length of ~8000 nt. Many studies reported that MALAT1 is a nuclear lncRNA variably expressed in many types of cancers such as endometrial, breast, cervical, and lung cancers [77,78,79,80]. The overexpression of MALAT1 is related to hyperproliferation and metastasis [81]. Recent studies show that MALAT1 regulates alternative RNA splicing of endogenous target genes using SR protein phosphorylation levels or by interacting with splicing factors such as serine-/arginine-rich splicing factor 1 (SRSF1) and SRSF3 [82]. It was demonstrated in basal-like breast cancers that oncogenic splicing factor SRSF1 bridges MALAT1 to mutant p53 and ID4 transcription factors [82]. mut-p53 and ID4 take away MALAT1 from nuclear speckles and favor its recruitment onto the chromatin. This enables aberrant binding of MALAT1 on VEGFA pre-mRNA and modulation of VEGFA isoforms expression [82]. 

Furthermore, MALAT1 overexpression has been reported to induce the deacetylation activity of SIRT1 and to decrease the p53 acetylation level. In this way, lncRNA suppresses the transcription levels of p53 target genes (p21, Bax, Puma, Stat3, Cyclin D, and Cyclin E), increasing the proliferation rate of the cells [83]. However, considering that many post-transcriptional regulatory mechanisms of wt-p53 have been found to also be common in mut-p53, we cannot exclude that even in MALAT1 overexpressed tumors, the mechanism of regulation of mut-p53 homeostasis expression can be conserved. 

Human pancreatic ductal adenocarcinoma (PDAC) is one of the most aggressive solid malignancies characterized by insensitivity to current therapy, metastasis, and poor prognosis, with a 5-year overall survival rate of less than 5% [84]. It has been shown that mut-p53 proteins promote tumor proliferation, invasion, metastasis, and chemoresistance in PDAC [85,86,87]. Interestingly, lincRNA-1611 was found to have significantly high expression in 22 out of 26 freshly resected human PDAC tissues, compared to normal pancreatic tissues, also displaying a positive correlation with mutations in the *TP53* gene (Table 3) [88]. These data suggested that lincRNA-1611 may be used as new biomarker of PDAC tumor progression and in the future as a potential target in the personalized medicine.

Head and neck squamous cell carcinomas (HNSCCs) arise in the mucosal linings of the upper aerodigestive tract, are heterogeneous in nature and present the highest levels of tumor immune infiltration among solid cancers [89]. DNA sequencing data have revealed that mutation in the *TP53* gene is frequent in HNSCC, occurring in up to 85% of human papillomavirus (HPV)-negative primary tumors, and where *TP53* mutations are associated with poor response to the radio- and/or chemo- therapies and decreased survival [50,90,91]. It was reported that mut-p53, forming oncogenic complexes with NF-Y and E2F1, binds to the *MIR205HG* gene promoter and positively regulates its transcription (Table 3). By splicing of MIR205HG pre-mRNA, two types of functionally-independent RNAs are produced—MIR205HG lncRNA and miR-205-5p [92].

In HNSCC cancer cells, MIR205HG binds and sequesters endogenous miR-590-3p leading to increased cyclin B, cdk1, and YAP protein expression and to the deregulated cell growth [92]. In HNSCC patients, high levels of lncMIR205HG are associated with tumoral samples that depend upon the expression of mut-p53 proteins [92], whereas although mut-p53 forms transcriptional oncogenic complexes with the YAP cofactor [18], it is evident that a type of feedback is established within a mut-p53/lncRNAs/miRNAs network for the homeostasis of pro-proliferative transcriptional complexes driven by mutant p53 in cancer cells.

Although other studies describe the existence of lncRNAs in relation to the expression of mutant forms of p53, their cellular function in cancer is still unknown.

## 4. Mutant p53 and Circular RNAs

The first circular RNAs are serendipitously discovered after 1991 in different organisms such as human, mouse, and monkey [93]. Despite intense studies, the evidence that these forms of RNA were translated was never found, thus raising questions about their function.

More recently, next-generation sequencing allows us to discover new non-coding RNA species expressed in eukaryotic cells and some computational algorithms can predict circular RNAs (circRNAs), which are most commonly found at back-splicing junctions [94,95]. circRNA is a type of single-stranded RNA generally formed by alternative splicing of pre-mRNA where the 5′ upstream splice acceptor is joined to a 3′ downstream splice donor in a process called ‘backsplicing’. This event forms covalently-closed continuous loops without polyadenylated tails and results in exceptionally stable molecules insensitive to ribonucleases [96]. 

CircRNAs are very stable molecules due to their circular structure and are not easily degraded by RNA nucleases. It was documented that they can act as miRNA sponges, as regulator of gene expression and transcription, and also as RNA-binding protein (RBP) sponges [96]. Through these mechanism circRNAs can affect such cancer cell activities as proliferation, migration, invasion and metastasis therefore becoming effective diagnostic and prognostic biomarkers and possible therapeutic targets in the precision anticancer therapy [95,96]. This potential, that renders circRNAs good candidates to be strong predictive biomarkers, is further substantiated by their detection in body fluids (blood, urine, and saliva) which is an essential feature in the liquid biopsy [96,97].

Despite our knowledge on the mechanisms that regulate the expression of these important RNAs, hundreds of circRNAs have been shown to cover an important role in several human cancers becoming key players in tumor pathogenesis and affecting several of the hallmarks of cancer such as proliferative signaling, epithelial-to-mesenchymal transition, angiogenesis, apoptosis, or drug resistance [98]. 

The first study that documented an existing crosstalk between mut-p53 and a circRNA was in head and neck squamous cell carcinoma (Table 4) [99]. In a cohort of 115 HNSCC patients, it was found that circPVT1 was over-expressed in tumoral tissues compared to matched non-tumoral tissues, with particular enrichment in patients carrying mutations in the *TP53* gene [99]. High circPVT1 expression was correlated with an increase of the malignant phenotype in HNSCC cell lines. With regard to the mechanism, circPVT1 expression is transcriptionally enhanced by the mut-p53/YAP/TEAD complex, hence circPVT1 acts as an oncogene regulating cell proliferation in HNSCC [99].

In breast cancer cells, it has been shown that wt-p53 enhanced circ-Ccnb1 expression, whereas repression of wt-p53 or the expression of mutated p53 repressed circ-Ccnb1 expression levels (Table 4) [100]. 

Analysis of circ-Ccnb1 levels in samples from breast cancer patients showed that the subgroup expressing mut-p53 proteins displayed significantly lower levels of circ-Ccnb1, compared to the p53 wild-type samples [100]. An in vivo xenograft experiment showed that the ectopic expression of circ-Ccnb1 significantly repressed tumor growth [100]. Therefore, circ-Ccnb1 can be considered a tumor suppressor that is down-regulated by mut-p53 proteins and which could be an excellent target that could be reactivated by targeted therapies. With regard to the mechanism, the circular RNA circ-Ccnb1 dissociates CyclinB1/Cdk1 mitotic complex suppressing cell invasion and tumorigenesis [101]. What has been said so far, is that mut-p53 directly promotes high proliferation, by inducing the transcription of the *ccnb1* and *cdk1* genes [16] and indirectly supporting the maintenance of the expression of its transcriptional cofactors such as YAP [18] and by inhibiting biomolecules that destabilize mitotic pathways [100,101].

## 5. Conclusions

Numerous ncRNAs are aberrantly modulated in diverse tumors and are often cancer- and stage-specific. Many ncRNAs or their processed products are stable in body fluids and detectable in the plasma and urine of cancer patients [102]. Their levels and presence could be suitable for better characterization of the kind of tumor, progression, and/or metastasization. All these factors render ncRNAs an attractive choice as non-invasive biomarkers and therapeutic targets for the treatment of cancer. Thanks to the recent success of RNAi-based and oligo-based drugs [33,34], several therapeutic clinical trials with ncRNAs have begun and are currently active and are potentially able to impact patient care (http://clinicaltrials.gov). 

Despite the increasing amount of knowledge on the pathways regulated by both wild-type and mutant p53 proteins, the crosstalk of signaling pathways dependent on non-coding RNA, wt, and mut-p53 is still an interesting field to elucidate. Recently, a multi-omic approach combining DNA interactome, transcriptome, and proteome analysis have brought out a “core” and specific properties common to the different mutant p53 proteins that suggests an influence of mut-p53 on the protein content of cancer cells that goes beyond the control of transcriptional mechanisms to the proteasome-mediated alteration of the proteome [103]. The induction of proteasome activities and expression were shown as a common program of diverse GOF mut-p53 proteins in cell lines and in a mutant p53 knock-in mouse lymphoma model [103]. These results hold therapeutic implications. The treatment of mut-p53 with the small molecule APR-246 in combination with the proteasome inhibitor carfilzomib showed a decrease in mutant p53-dependent primary tumor growth and eradication of metastasis in mouse TNBC xenograft assays [103].

Based on these premises, applying combined treatments which target both the mutant p53 proteins and the pathways modulated by the non-coding RNAs is warranted, and in a short time too.

## Figures and Tables

**Table 1:** MicroRNAs up-regulated by Gain of Function mut-p53.

microRNA Name	Cancer	References
miR-155	breast	[47]
miR-128-2	breast	[48]
miR-205-5p	head and neck	[44,49]
miR-1246	colon	[55]

**Table 2:** MicroRNAs down-regulated by gain of function (GOF) mut-p53.

microRNA Name	Cancer	References
miR-34	ovary	[57]
miR-34	lung	[58,59]
miR-223	breast, colon	[62]
miR-223-3p	lung	[63]
let-71	breast	[64]

**Table 3:** Long non coding RNAs regulated by gain of function mut-p53.

lncRNA Name	Cancer	References
lincRNA-p21 (wt-p53)	lung	[74]
MALAT1	breast	[82]
lincRNA-1611	pancreas	[88]
MIR205HG	head and neck	[92]

**Table 4:** Circular RNAs regulated by Gain of Function mut-p53.

circRNA Name	Cancer	References
circPVT1	head and neck	[98]
Circ-Ccnb1	breast	[99,100]
